# Microvascular and Prognostic Effect in Lesions With Different Stent Expansion During Primary PCI for STEMI: Insights From Coronary Physiology and Intravascular Ultrasound

**DOI:** 10.3389/fcvm.2022.816387

**Published:** 2022-03-09

**Authors:** Xida Li, Shuo Sun, Demou Luo, Xing Yang, Jingguang Ye, Xiaosheng Guo, Shenghui Xu, Boyu Sun, Youti Zhang, Jianfang Luo, Yingling Zhou, Shengxian Tu, Haojian Dong

**Affiliations:** ^1^Guangdong Provincial People's Hospital Zhuhai Hospital (Zhuhai Golden Bay Hospital), Zhuhai, China; ^2^Department of Cardiology, Southern Medical University, Guangzhou, China; ^3^Guangdong Provincial People's Hospital, Guangdong Cardiovascular Institute, Guangdong Academy of Medical Sciences, Guangzhou, China; ^4^The First Affiliated Hospital of Guangzhou Medical University, Guangzhou, China; ^5^Department of Cardiology, Guangdong Provincial Jiexi People's Hospital, Jiexi, China; ^6^Med-X Research Institute, School of Biomedical Engineering, Shanghai Jiao Tong University, Shanghai, China

**Keywords:** ST-elevation myocardial infarction (STEMI), stent expansion, microcirculation, major adverse cardiovascular events (MACEs), IVUS

## Abstract

**Background:**

While coronary stent implantation in ST-elevation myocardial infarction (STEMI) can mechanically revascularize culprit epicardial vessels, it might also cause distal embolization. The relationship between geometrical and functional results of stent expansion during the primary percutaneous coronary intervention (pPCI) is unclear.

**Objective:**

We sought to determine the optimal stent expansion strategy in pPCI using novel angiography-based approaches including angiography-derived quantitative flow ratio (QFR)/microcirculatory resistance (MR) and intravascular ultrasound (IVUS).

**Methods:**

*Post-hoc* analysis was performed in patients with acute STEMI and high thrombus burden from our prior multicenter, prospective cohort study (ChiCTR1800019923). Patients aged 18 years or older with STEMI were eligible. IVUS imaging, QFR, and MR were performed during pPCI, while stent expansion was quantified on IVUS images. The patients were divided into three subgroups depending on the degree of stent expansion as follows: overexpansion (>100%), optimal expansion (80%−100%), and underexpansion (<80%). The patients were followed up for 12 months after PCI. The primary endpoint included sudden cardiac death, myocardial infarction, stroke, unexpected hospitalization or unplanned revascularization, and all-cause death.

**Results:**

A total of 87 patients were enrolled. The average stent expansion degree was 82% (in all patients), 117% (in overexpansion group), 88% (in optimal expansion), and 75% (in under-expansion). QFR, MR, and flow speed increased in all groups after stenting. The overall stent expansion did not affect the final QFR (*p* = 0.08) or MR (*p* = 0.09), but it reduced the final flow speed (−0.14 cm/s per 1%, *p* = 0.02). Under- and overexpansion did not affect final QFR (*p* = 0.17), MR (*p* = 0.16), and flow speed (*p* = 0.10). Multivariable Cox analysis showed that stent expansion was not the risk factor for MACE (hazard ratio, HR = 0.97, *p* = 0.13); however, stent expansion reduced the risk of MACE (HR = 0.95, *p* = 0.03) after excluding overexpansion patients. Overexpansion was an independent risk factor for no-reflow (HR = 1.27, *p* = 0.02) and MACE (HR = 1.45, *p* = 0.007). Subgroup analysis shows that mild underexpansion of 70%−80% was not a risk factor for MACE (HR = 1.11, *p* = 0.08) and no-reflow (HR = 1.4, *p* = 0.08); however, stent expansion <70% increased the risk of MACE (HR = 1.36, *p* = 0.04).

**Conclusions:**

Stent expansion does not affect final QFR and MR, but it reduces flow speed in STEMI. Appropriate stent underexpansion of 70–80% does not seem to be associated with short-term prognosis, so it may be tolerable as noninferior compared with optimal expansion. Meanwhile, overexpansion and underexpansion of <70% should be avoided due to the independent risk of MACEs and no-reflow events.

## Introduction

Primary percutaneous coronary intervention (pPCI), especially coronary stent implantation, is one of the most important treatments of ST-elevation myocardial infarction (STEMI). Complications associated with stents have been noticed. Previous studies have shown that stent underexpansion diagnosed by intravascular imaging, such as intravascular ultrasound (IVUS), is associated with stent thrombosis and other major adverse cardiovascular events (MACEs) (1). Underexpansion usually results from calcification, fibrotic lesions, inappropriate size of stent, or insufficient pressure of dilation. Postdilation with a high-pressure balloon is one of the most common solutions of underexpansion. However, postdilation of a stent with high pressure in a culprit lesion with a heavy thrombus burden increases the risk of distal embolism for no-reflow phenomenon in STEMI (2). The relationship between stent expansion and vascular function and prognosis of pPCI in STEMI patients is rarely reported.

Quantitative flow ratio (QFR), as a novel angiography-based approach, has been recently reported to provide the physiological value of the accuracy of detection of vessel dysfunction and risk from cardiovascular events (3). In the latest results of the FAVOR III China study, a QFR-guided PCI strategy was proved to be able to reduce major cardiac events compared with standard angiography guidance PCI strategy (4). By focusing on geometric and functional outcomes of stenting in STEMI, we aimed to explore the optimal criteria for adequate stent expansion for post-pPCI prognosis by IVUS and QFR.

## Methods

This study was a subgroup analysis of a multicenter, open-label, prospective cohort study (the outcomes in STEMI patients with high thrombus burden treated by deferred vs. immediate stent implantation in primary percutaneous coronary intervention: a prospective cohort study, registered at www.chictr.org.cn, ChiCTR1800019923), which was conducted in three cardiovascular centers in South China (Guangdong Provincial People's Hospital, Guangzhou City; Guangdong Provincial People's Hospital Zhuhai Hospital, Zhuhai City; and Jiexi County People's Hospital, Jiexi City) from January 2018 to January 2020. The research was approved by the Ethics Committee of Guangdong Provincial People's Hospital, Guangzhou, China, and the number of the Medical ethics approval document was [GDREC2018346H (R2)]. All enrolled patients with successful stenting and complete IVUS and QFR analysis were included. Patients with incomplete data, disqualified images of IVUS or QFR, cardiogenic shock, or chronic kidney disease of stage 5 were excluded. This study was approved by the institutional review board, and written informed consents were obtained from all the patients. The patients were premedicated with 300 mg of aspirin and 300 mg of clopidogrel or 180 mg of ticagrelor. Antithrombotic treatments, including heparin or glycoprotein IIb/IIIa inhibitors, were administered at the operator's discretion. Aspirin 100 mg/day and clopidogre l75 mg/day or ticagrelor 90 mg/day were prescribed for 12 months. A data center collected clinical and laboratory data from medical records and telephone interview follow-up after hospitalization. MACEs were defined as sudden cardiac death, myocardial infarction, stroke, unexpected hospitalization or unplanned revascularization, and all-cause death. All the clinical events were adjudicated by an independent and professional event adjudication committee. The patients were followed up for 12 months.

### IVUS Imaging and Analysis

**Figure 1:**
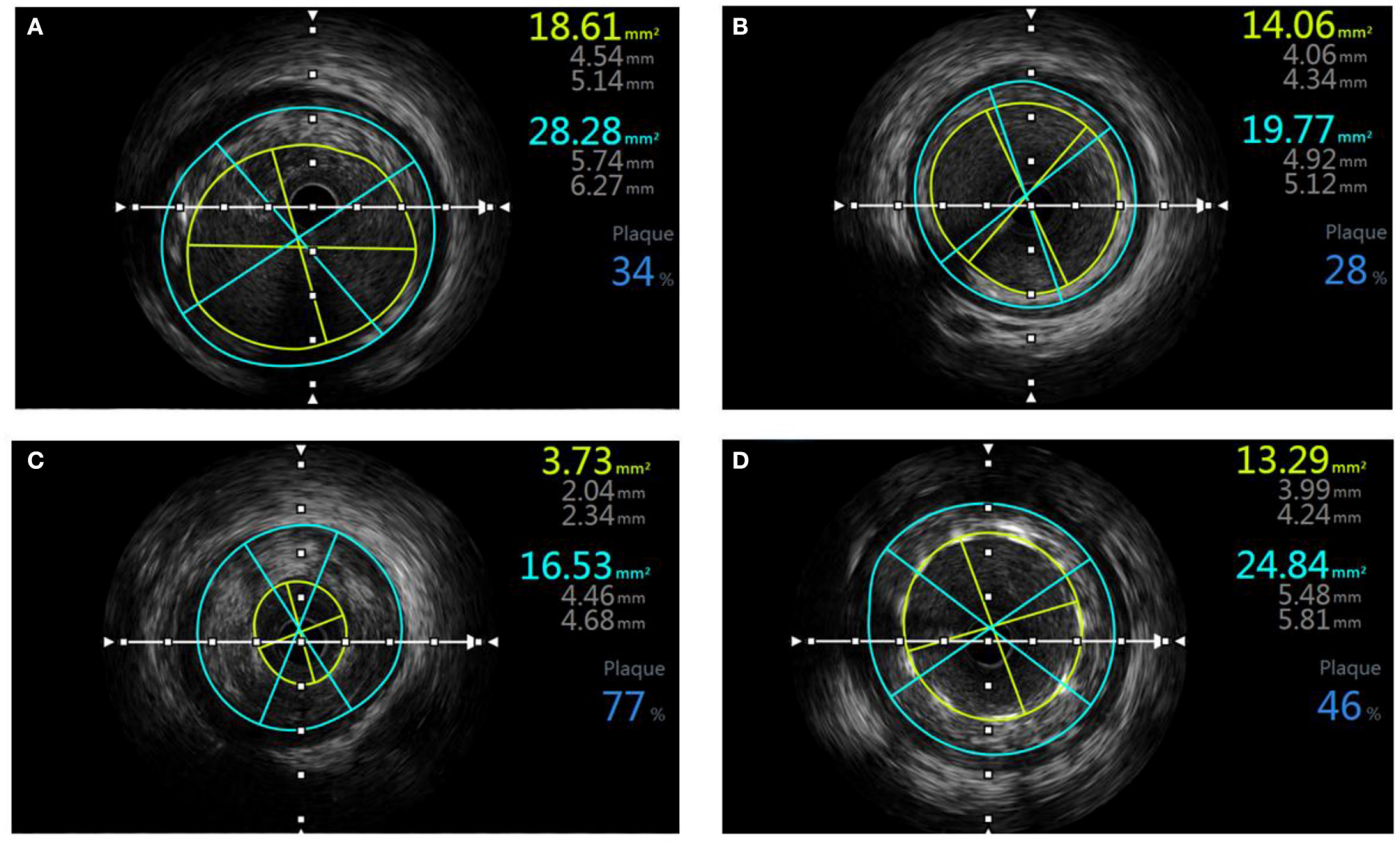
An example of IVUS imaging and analysis of stent expansion. **(A)** EEM CSA and lumen CSA of proximal normal-looking segment; **(B)** EEM CSA and lumen CSA of distal normal-looking segment; **(C)** EEM CSA and lumen CSA of minimal culprit segment; **(D)** EEM, CSA, and MSA after stenting, the stent expansion is 13.29/[(14.06+18.61)/2] x 100 = 82.4%. EEM, external elastic membrane; CSA, crosssectional area; MSA, minimum stent cross-sectional area.

**Figure 2:**
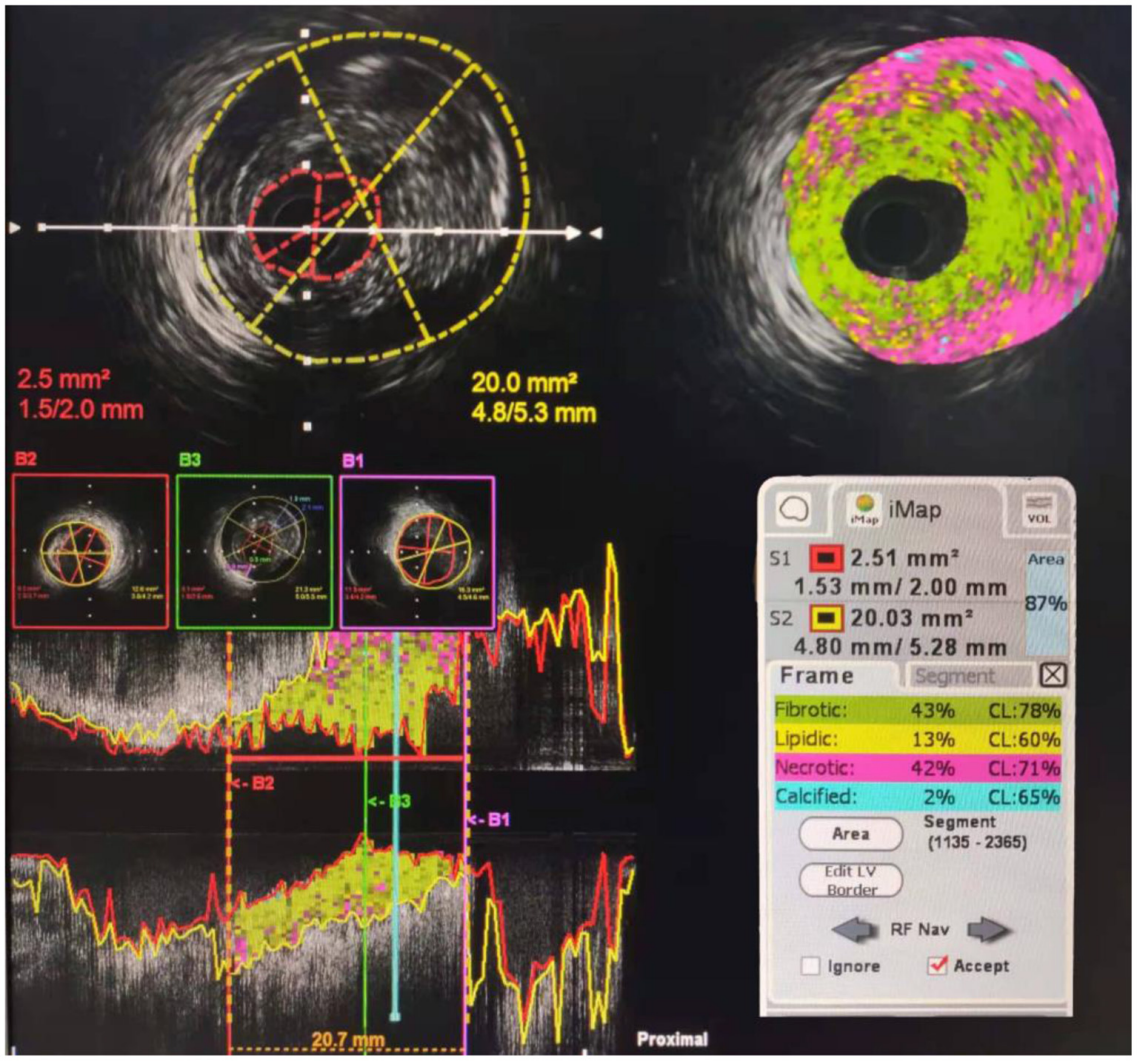
An example of plaque analysis. The ratio of plaque is fibrotic: 43%, lipidic: 13%, necrotic: 42%, and calcified: 2% representatively.

Intravascular ultrasound was performed with a commercially available mechanical sector scanner (Boston Scientific, Natick, Massachusetts), incorporating a 40-MHz single-element beveled transducer rotating at 1,800 rpm. The ultrasound catheter was advanced at least 10 mm beyond the stent, and an imaging run was performed to a point at least 10 mm proximal to the stent using a motorized transducer pullback at 0.5 mm/s. Data were recorded during the pullback. All IVUS images were analyzed by an independent experienced technician. Minimum stent crosssectional area (MSA), proximal and distal reference segment external elastic membrane (EEM), and lumen crosssectional area (CSA) were measured. The proximal and distal reference segments were measured at the normal-looking crosssections within 5 mm proximal or distal to the stent. The stent expansion was calculated as follows: stent expansion = MSA in stent/mean of proximal and distal reference CSA, where the mean of reference lumen CSA = (proximal CSA+ distal CSA)/2. Stent expansion was classified as underexpansion (<80%), optimal expansion (80–100%), and overexpansion (>100%), as shown in Figure 1. Then the plaque was analyzed. Plagues were divided into four types: fibrotic, lipidic, necrotic, and calcified. The ratio of every type of plaque was calculated as shown in Figure 2.

### 3D-QCA Reconstruction

Two angles of end-diastolic angiographic projections that were more than 25° apart, where there was no foreshortening or overlapping of the segment, were captured. This was carried out using established and well-validated software. In the obtained images, the lesion length and minimum lumen diameter (MLD) were analyzed.

### QFR and MR Analysis

**Figure 3:**
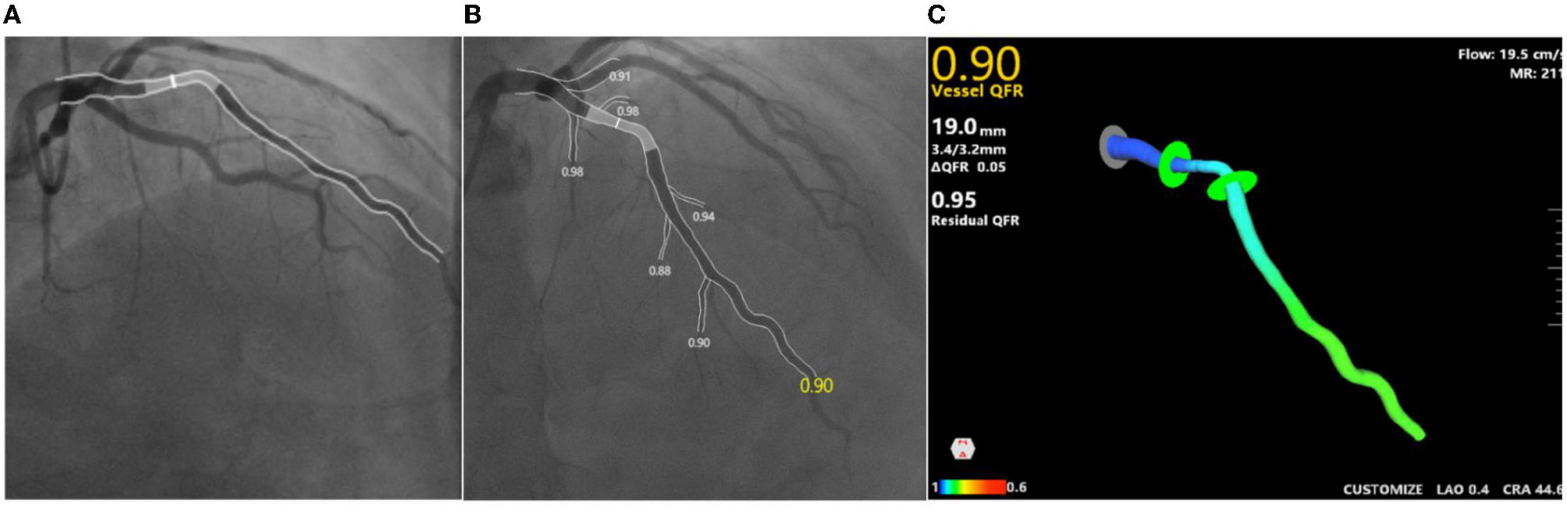
An example of QFR and MR analysis. **(A,B)** Angiographs of two angles of one same culprit lesion; **(C)** cQFR is 0.9, MR is 211mm Hg*s/m, flow speed is 19.5cm/s. cQFR, contrast-flow quantitative flow ratio; MR, microcirculatory resistance.

Quantitative flow ratio, MR, and flow speed were measured immediately after pretreatment with percutaneous transluminal coronary angioplasty (POBA) or thrombus aspiration and after stent implantation. QFR was computed with 3D/QFR software using two approaches: (1) assuming a fixed blood flow (fQFR); (2) taking into account the flow velocity estimated by the time needed for the contrast agent to fill the segment, which is known as contrast-flow QFR (cQFR) (4–9). Then, angiography-derived microcirculatory resistance (MR) was computed by Angioplus System, as shown in Figure 3. Traditional FFR requires the vessels to be in a hyperemic state, and the QFR system calculates the QFR and MR parameters of vessels without hyperemic state through an algorithm that has been proved to be consistent with FFR (6). Next, cQFR–fQFR was calculated, which represented microvascular dysfunction (MVD). The ΔQFR, ΔMR, and Δflow speed were calculated as the difference between the parameters after stenting and those before stenting. MR deterioration was defined as MR increase after stenting. Eligible patients received offline QFR evaluation by two experienced technicians (Shanghai Jiao Tong University-Pulse Medical Imaging Technology Joint Laboratory, Shanghai, China) blinded to the study, and computation of QFR was performed using a prototype software (AngioPlus Core, Pulse Medical Imaging Technology, Shanghai, China).

### Statistical Analysis

Numerical variables were presented as medians, whereas categorical variables were shown as absolute values and percentages. Comparisons between numerical variables were performed using analysis of variance (ANOVA), whereas categorical variables were compared using the chi-square test. Comparison of parameters before and after stenting was performed using the paired-samples *t*-test. For underexpansion vs. optimal-expansion vs. overexpansion, under-expansion was the reference. Normality was tested prior to the ANOVA test in parametric data, and if the normality test fails, a nonparametric ANOVA should be used. Linear regression analyses were used to identify the relationship and affection of stent expansion and Cox survival regression was used to identify the risk factors, QFR predictor, and MACEs. Statistical analysis was performed by SPSS (version 23; IBM Corp, Armonk, NY, USA). A *p*-value of 0.05 was considered significant.

## Results

### Baseline and QCA Characteristics

**Figure 4:**
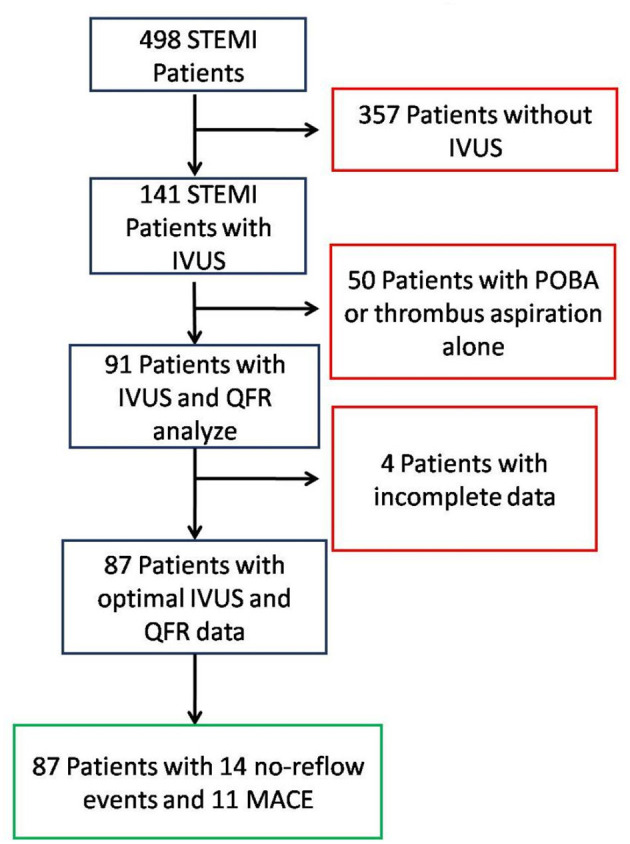
Flowchart of study inclusion and exclusion.

During the 1-year study period, 498 STEMI patients underwent pPCI. Among them, 141 patients were treated with IVUS guidance and QFR analysis; 50 were excluded due to POBA or thrombus aspiration alone; and five were excluded due to incomplete data, as shown in Figure 4. A total of 87 patients were finally included. Of those 87 patients, seven had no-reflow events and 11 had MACE (one cardiac death, one stroke, three cases of myocardial infarction, and six cases of heart failure), as shown in Table 1. Among the 11 patients with MACEs, 10 were hospitalized and eight underwent revascularization. Baseline demographic and QCA data are shown in Table 1. The average stent expansion degree in all patients, over-expansion, optimal expansion, and underexpansion groups were 82, 117, 88, and 75%, respectively. The over-expansion group had a higher rate of MACEs (*p* < 0.05). Lumen CSA of reference (overexpansion group 6.1 ± 2.6 mm^2^ vs. optimal expansion group 9.0 ± 3.2 mm^2^ vs. underexpansion group, 10.7 ± 4.1 mm^2^, *p* = 0.02), rate of no-reflow event (over 2, 40% vs. optimal 2, 6.6% vs. under 3, 5.8%, *p* = 0.01), and rate of MACE (over-expansion group: 3, 60% vs. optimal expansion group: 1, 3.3% vs. under-expansion group: 7, 13.7%, *p* = 0.01) are different among the patients with different stent expansion degrees, and other characteristics had no significant difference.

**Table 1:** Baseline demographic, QCA, and IVUS characteristics of the patients.

	**Studied patients)**	**Over-expansion**	**Optimal expansion**	**Under-expansion**	***p***
	**(*n =* 87)**	**(*n =* 5)**	**(*n =* 30)**	**(*n =* 52)**	
Age (years)	58.1	59.8	54.2	59.6	0.18
Gender (male)	56 (64%)	3 (60%)	26 (86%)	27 (52%)	0.21
BMI	25.2	24.2	25.0	25.4	0.12
Current smoker	61 (71%)	2 (40%)	22 (73%)	37 (72%)	0.35
Diabetes mellitus	10 (11%)	0 (0%)	4 (21%)	6 (11%)	0.19
Hypertension	51 (59%)	3 (60%)	19 (63%)	29 (57%)	0.13
Cr	96.3	75.4	74.8	100.5	0.26
LDLC	3.31	3.29	3.61	3.3	0.21
Target coronary artery						
Left anterior descending artery	42 (49%)	2 (40%)	16 (53%)	24 (47%)	0.14
Left circumflex artery	17 (20%)	1 (20%)	5 (17%)	11 (21%)	0.11
Right coronary artery	27 (31%)	2 (40%)	9 (30%)	16 (31%)	0.18
TIMI flow (before stenting)						
0–1 (%)	57 (66%)	3 (60%)	16 (53%)	38 (75%)	0.22
2 (%)	7 (8.1%)	1 (20%)	3 (10%)	5 (10%)	0.27
3 (%)	21 (24.4%)	1 (20%)	11 (37%)	9 (25%)	0.21
Angiographic analyses						
Reference vessel diameter (mm)	3.5 ± 0.8	3.0 ± 0.8	3.5 ± 0.9	3.6 ± 0.7	0.07
MLD (mm)	1.3 ± 0.9	1.1 ± 0.6	0.9 ± 0.7	1.4 ± 0.5	0.09
Lesion length (mm)	17 ± 10	21 ± 14	17 ± 15	16 ± 6	0.14
IVUS analyses						
Reference (normal-looking segment)						
Lumen CSA (mm^2^)	9.9 ± 4.0	6.1 ± 2.6	9.0 ± 3.2	10.7 ± 4.1	0.02
EEM CSA (mm^2^)	14.1 ± 5.3	10.1 ± 4.2	14.6 ± 4.5	14.4 ± 5.6	0.06
Reference (minimum lumen segment)						
Lumen CSA (mm^2^)	3.5 ± 2.6	4.0 ± 2.7	4.1 ± 4.3	3.3 ± 1.5	0.11
EEM CSA (mm^2^)	14.9 ± 5.0	10.3 ± 4.4	14.2 ± 5.7	15.8 ± 4.5	0.23
Plaque burden (%)	79 ± 5	75 ± 5	81 ± 4	79 ± 6	0.18
Plaque ratio (%)						
Fibrotic	65%	64%	67%	65%	0.51
Necrotic	24%	26%	24%	23%	0.04
Lipidic	9.6%	9%	9.6%	9%	0.12
Calcified	1.4%	1%	2.4%	3%	0.01
Plaque volume (mm3)						
Fibrotic	6.66 ± 2.2	6.34 ± 2.4	5.75 ± 1.9	6.89 ± 2.6	0.39
Necrotic	2.5 ± 1.1	2.85 ± 0.6	2.57 ± 1.2	2.41 ± 1.3	0.04
Lipidic	0.99 ± 0.02	1.05 ± 0.03	0.9 ± 0.01	1.01 ± 0.02	0.07
Calcified	0.15 ± 0.016	0.11 ± 0.012	0.14 ± 0.011	0.23 ± 0.019	0.02
Stent segment						
Stent diameter (mm)	3.2 ± 0.7	3.0 ± 0.4	3.3 ± 0.4	3.1 ± 0.8	0.15
Stent length (mm)	25 ± 7	28 ± 5	24 ± 5	25 ± 8	0.27
Stent MLD (mm)	2.7 ± 0.5	2.5 ± 0.5	2.8 ± 0.5	2.6 ± 0.5	0.14
Maximum inflation pressure (atm)	16.8 ± 5.2	17.1 ± 4.5	16.7 ± 4.8	16.20 ± 5.0	0.09
Minimum stent CSA (mm^2^)	6.9 ± 2.4	6.4 ± 3.0	8.0 ± 2.4	6.5 ± 2.2	0.10
Stent expansion (%)	82 ± 24	117 ± 7	88 ± 19	75 ± 15	0.01
MR deterioration after stenting, n (%)	37 (42.5%)	3 (60%)	14 (46.7%)	20 (38.4%)	0.04
No-reflow	7 (8.1%)	2 (40%)	2 (6.6%)	3 (5.8%)	0.01
MACE	11 (12.7%)	3 (60%)	1 (3.3%)	7 (13.7%)	0.01
EF (%)	50.9	55.2	53.6	50.2	0.25
COST (¥)	58,100.5	67,045.64	47,872.38	58,113.19	0.11

*BMI, body mass index; Cr, creatinine; LDLC, low density lipoprotein cholesterol; CSA, cross-sectional area; EEM, external elastic membrane; MLD, minimum lumen diameter; IVUS, intravascular ultrasound; MACE, major adverse cardiovascular events; EF, ejection fraction*.

### QFR and IVUS Characteristics

**Figure 5:**
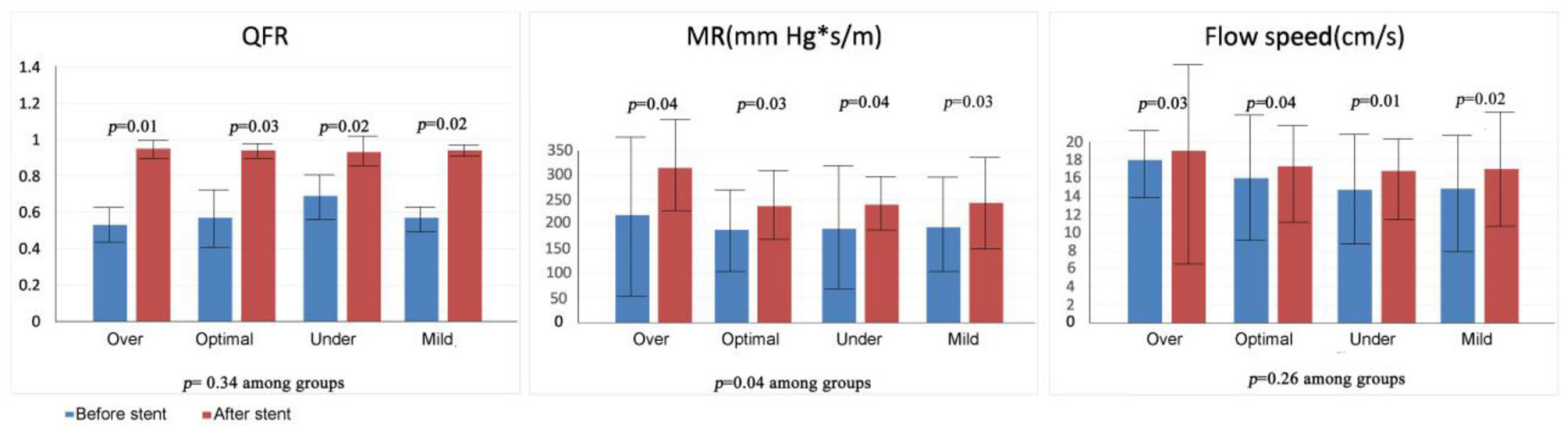
Data of QFR characters.

Quantitative flow ratio findings are presented in Tables 2, 3 and are depicted in Figure 5. Patients with stent expansion of 70–80% were classified as a subgroup of mild-underexpansion and compared with the other three groups. QFR increased significantly after stenting (over-expansion group: 0.53 vs. 0.95; optimal expansion group: 0.57 vs. 0.94; underexpansion group: 0.69 vs. 0.93, *p* < 0.05), with no significant difference among the groups (*p* > 0.05). The overexpansion group had a higher MR before stenting (overexpansion group 219 mm Hg^*^s/m vs. optimal expansion group 189 mm Hg^*^s/m vs. underexpansion group 191 mm Hg^*^s/m, *p* < 0.05). The MR before stenting was different among the groups (overexpansion group 219 mm Hg^*^s/m vs. optimal expansion group 189 mm Hg^*^s/m vs. underexpansion group 191 mm Hg^*^s/m, *p* < 0.05). MR increased in all the groups after stenting (overexpansion group 315 mm Hg^*^s/m vs. optimal expansion group 237 mm Hg^*^s/m vs. underexpansion group 240 mm Hg^*^s/m, *p* > 0.05 among groups), and ΔMR differed among the groups (over-expansion group 87 mm Hg^*^s/m vs. optimal expansion group 38 mm Hg^*^s/m vs. underexpansion group 52 mm Hg^*^s/m, *p* = 0.04). Flow speed increased significantly after stenting (overexpansion group: 18.2 cm/s vs. 19.1 cm/s; optimal expansion group: 16.0 vs. 17.3; underexpansion group: 14.7 vs. 16.8, p < 0.05), with no significant difference among the groups (p > 0.05). cQFR–fQFR differed among the groups before stenting (overexpansion group 0.16 vs. optimal expansion group 0.008 vs. underexpansion group 0.041, *p* = 0.04), but did not differ among the groups after stenting (overexpansion group 0.06 vs. optimal expansion group 0.01 vs. underexpansion group 0.003, *p* > 0.05). ΔcQFR–fQFR differed among the groups (overexpansion group −0.07 vs. optimal expansion group −0.01 vs. underexpansion group − 0.04, *p* = 0.02). Subgroup analysis showed that the MR deterioration group had significantly higher stent expansion (95.6 vs.79.8%, *p* < 0.01), higher MR (246.1 vs. 157.2, *p* < 0.01), and higher flow speed (15.9 vs. 14.9, *p* < 0.01) before stenting, as well as higher MR (287.7 vs. 197.8, *p* < 0.01) and lower flow speed (17.1 vs. 18.1, *p* < 0.01) after stenting than the MR improvement group. In 68 (78.1%) patients, plaque data were available. The following four types of plaque were found in our study: fibrotic, lipidic, necrotic, and calcified. We analyzed the data and found that the volume and ratio of necrotic plaque were higher in the overexpansion group, while the volume and ratio of calcified plaque were higher in the underexpansion group.

**Table 2:** QFR characteristics.

	**Studied patients**	**Over-expansion**	**Optimal-expansion**	**Under-expansion**	**Mild-Under-expansion**	***p***
	**(*n =* 87)**	**(*n =* 5)**	**(*n =* 30)**	**(*n =* 52)**	**(*n =* 34)**	
cQFR before stenting	0.64	0.53	0.57	0.69	0.57	0.09
MR before stenting (mm Hg*s/m)	203.9	219.3	189.4	191.5	198.3	0.02
cQFR–fQFR before stenting	0.043	0.16	0.008	0.041	0.036	0.04
Flow speed before stenting (cm/s)	15.6	18.2	16.0	14.7	14.9	0.17
cQFR after stenting	0.93	0.95	0.94	0.93	0.94	0.64
MR after stenting (mm Hg*s/m)	268.5	315.1	237.5	240.4	247.0	0.24
cQFR–fQFR after stenting	0.004	0.06	0.001	0.003	0.007	0.08
Flow speed after stenting (cm/s)	17.4	19.1	17.3	16.8	17.1	0.28
ΔQFR	0.32	0.4	0.4	0.2	0.24	0.31
ΔMR (mm Hg*s/m)	59	87	38	52	48	0.04
ΔcQFR–fQFR	−0.05	−0.07	−0.01	−0.04	−0.02	0.02
ΔFlow speed (cm/s)	0.54	0.78	1.03	0.91	0.99	0.39

**Table 3:** Subgroup analyses of MR deterioration.

	**Studied patients**	**MR deterioration**	**MR improvement**	***p***
	**(*n =* 87)**	**(*n =* 37)**	**(*n =* 50)**	
QCA stenosis rate (%)	90	95	87	0.03
Reference lumen CSA (mm^2^)	9.9	8.6	11.2	0.01
cQFR before stenting	0.64	0.63	0.65	0.25
MR before stenting (mm Hg*s/m)	203.9	246.1	157.2	0.005
Flow speed before stenting (cm/s)	15.6	15.9	14.9	0.004
cQFR after stenting	0.93	0.90	0.93	0.28
MR after stent (mm Hg*s/m)	268.5	287.7	197.8	0.04
Flow speed after stenting (cm/s)	17.4	17.1	18.1	0.04
Stent expansion (%)	82.1	95.6	79.8	0.001

### Regression Analysis

**Figure 6:**
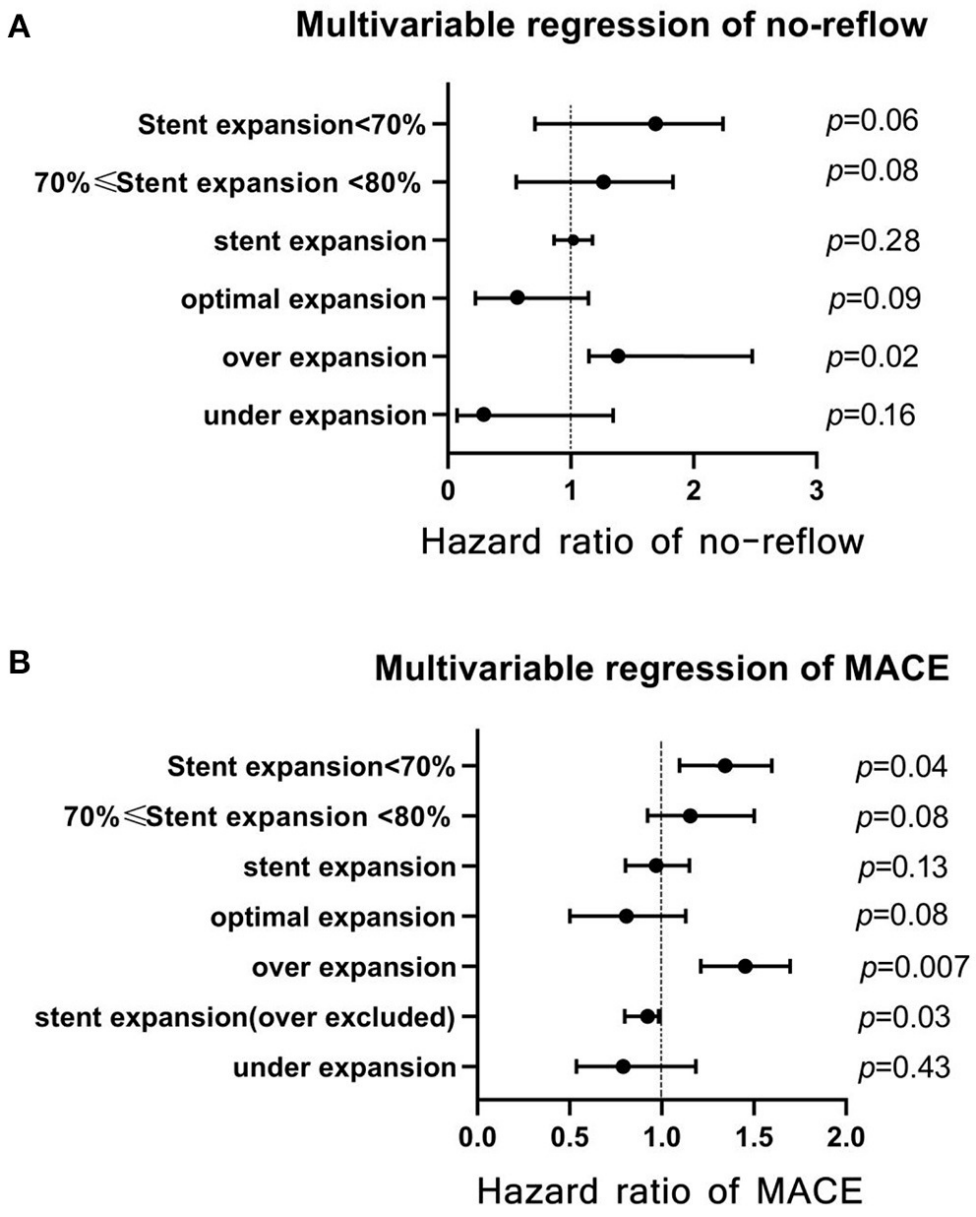
Multivariate Cox regression analyses of stent expansion for prognosis. **(A)** multivariate regression of no-reflow; **(B)** multivariate regression of MACE. MACE, major cardiac adverse events.

The results of multivariate regression analysis are presented in Tables 4, 5 and are depicted in Figure 6. They showed that stent expansion did not affect QFR (*p* = 0.86) and MR (*p* = 0.10); however, it reduced the flow speed (−3.14 m/s per 1%, *p* < 0.01) after stenting. Under- and overexpansion were not associated with the change of QFR and MR after stenting (p > 0.05). Cox regression showed that expansion was not the risk factor for MACE (hazard ratio, HR = 0.97, *p* = 0.13) and no-reflow (HR = 1.02, *p* > 0.05). Additional analysis after excluding patients with overexpansion showed that stent expansion was the protective factor for MACE (HR = 0.95, *p* = 0.03) after excluding over-expansion patients. Over-expansion was the independent risk factor for no-reflow (HR = 1.27, *p* = 0.02) and MACE (HR = 1.45, *p* = 0.007). Asymptotic analysis showed that mild underexpansion of 70%−80% was not the risk factor for MACE (HR = 1.11, *p* = 0.08) and no-reflow (HR = 1.4, *p* = 0.08); however, the risk of MACE increased significantly when stent expansion was <70% (HR = 1.36, *p* = 0.04).

**Table 4:** Multivariate analyses of stent expansion of QFR.

**Multivariate analysis**	**HR**	***p***	**Confidence interval**
**Stent expansion (per 1% increase)**			
QFR after stenting	0.02	0.08	0.01–0.03
MR after stenting (mm Hg*s/m)	0.86	0.09	−0.7–1.8
Flow speed after stenting (cm/s)	−0.14	0.02	−0.81–0.10
**Optimal-expansion**			
QFR after stenting	0.27	0.24	0.08–0.52
MR after stenting (mm Hg*s/m)	39.1	0.26	−35–104
Flow speed after stenting (cm/s)	0.52	0.27	−0.42–0.76
**Over-expansion**			
QFR after stenting	0.31	0.17	−0.10–0.64
MR after stenting (mm Hg*s/m)	32.7	0.16	−28–231
Flow speed after stenting (cm/s)	0.41	0.10	−0.19–3.4
**Under-expansion**			
QFR after stenting	0.29	0.22	−0.06–0.63
MR after stenting (mm Hg*s/m)	40.5	0.31	−32–119
Flow speed after stenting (cm/s)	0.74	0.19	−0.67–1.57

**Table 5:** Multivariate cox survival regression analyses of stent expansion for prognosis.

	**HR**	***p***	**Confidence interval**
**Stent expansion (per 1%)**			
MACE	0.97	0.13	0.83–1.16
MACE (over-expansion excluded)	0.95	0.03	0.82–0.98
No-reflow	1.02	0.28	0.87–1.21
**Optimal-expansion**			
MACE	0.75	0.08	0.50–1.12
No-reflow	0.59	0.09	0.48–1.19
**Under-expansion**			
MACE	0.82	0.43	0.55–1.20
No-reflow	0.35	0.16	0.04–1.34
**Over-expansion**			
MACE	1.45	0.007	1.22–1.56
No-reflow	1.27	0.02	1.20–2.52
**70%≤stent expansion <80%**			
MACE	1.11	0.08	0.93–1.51
No-reflow	1.43	0.08	0.58–1.67
**Stent expansion <70%**			
MACE	1.36	0.04	1.09–1.58
No-reflow	1.74	0.06	0.73–2.28

## Discussion

This retrospective observational study was the first to evaluate the effects and optima of stent expansion in STEMI patients using a combination of coronary physiology and intracoronary imaging. QFR was introduced to assess the physiological function of coronary artery lesions as a noninvasive alternative to fractional flow reserve (FFR) (9–11). Studies have shown that QFR has an inverse relationship with prognosis (11–13) and consistency with FFR in patients with acute coronary syndrome (ACS), which emphasized the value of QFR in distinguishing potential ischemic lesions (9, 11, 14). The prognostic value of QFR has also been supported by IVUS parameters, which coronary imaging techniques use in predicting prognosis (14). MR calculated by QFR indicators reflects that vascular resistance is higher in the ischemic artery, which suggests that MR is a competitive tool for the evaluation of coronary microcirculation and effect of primary PCI (15). We analyzed the data of intravascular imaging, QFR, MR, and flow speed parameters, and found that stent expansion affected the flow speed but not the QFR and MR; appropriate underexpansion did not led to a significant risk of no-reflow and MACE. In contrast, overexpansion was associated with higher microvascular resistance, lower flow speed, and higher risk of no-reflow and MACE.

Stent expansion has been studied since the application of coronary stents (16, 17) and evaluated by imaging techniques, such as QCA, IVUS, and OCT. Early studies of intravascular imaging and vascular physiology have shown that underexpansion of pPCI may cause long-term lumen loss and stent thrombosis, leading to an increased risk of MACE, including myocardial infarction, heart failure, and cardiac death (1). The factors leading to underexpansion include small balloons, calcification, and inadequate dilation pressure, and are becoming fewer and fewer with the development of medical materials science. In this study, researchers observed less pressure of postdilation in elderly patients, fewer calcified vessels, and a high thrombotic burden. More positive dilation may be performed in younger patients, with fewer calcified vessels and a low thrombotic burden. The operators may give post-dilation as much as possible when underexpansion occurred, and then IVUS was performed to obtain the final image. Meanwhile, when the no-reflow occurred, an aggressive dilation strategy should not be taken. Optimal stent expansion gained by postdilation, which is related to a better prognosis, has been confirmed by multiple studies and included in the PCI recommendation consensus (18–21). However, overexpansion or oversize of stent caused by excessive postdilation has been increasing recently, but it has been less studied. A study showed that treatment of small vessels with smaller stents caused poor prognosis, and stent overexpansion, which is usually caused by big balloons or excessive dilation pressure decreased restenosis and target lesion revascularization rates, suggesting that over-expansion and larger stents may optimize outcomes in elective PCI patients (21). However, the safety, effects on vascular function, and long-term benefits of stent postdilation and overexpansion in STEMI patients remain unclear. A retrospective cohort study showed that MACEs did not significantly differ between the STEMI patients who underwent postdilation and those who had not, while the device-oriented composite endpoint (DOCE), including target vessel revascularization and cardiac death, was reduced by over half in the postdilation group, suggesting that operator-determined postdilation may improve prognosis in STEMI (22). Meanwhile, another study found that patients undergoing selective PCI with overexpansion had significantly greater in-scaffold luminal volume loss detected by optical coherence tomography at 6 months follow-up (23). However, these studies did not comprehensively analyze patients with optimal, under-, or overexpansion and selective or emergency PCI. The relationship between underexpansion, overexpansion, and vessel physiology or hemodynamics has not been further analyzed. In this study, we observed that the overexpansion group had smaller reference lumen CSA, which indicates that smaller vessels are more likely to be overexpanded; so, stent diameter should be carefully considered in smaller vessels. We also found that stent expansion <70% was the independent risk factor for poor prognosis, which is consistent with the previous consensus that residual stenosis segments are related with complications, such as in-stent thrombus; thus, optimal expansion is recommended (1). The conflicting results that stent expansion did not affect MACE or no-reflow overall, but did reduce the risk of MACE when overexpansion patients were excluded may be explained by the hypothesis that the protective effect of stent expansion has an optimum interval, while both the increase and the decrease in relation to this range may lead to the weakening of the protective effect. We found that overexpansion was an independent risk factor for MACE and no re-flow, which is meaningful to the procedure of PCI and similar to the conclusion of another study that overexpansion in patients with acute myocardial infarction is related to a higher incidence of no-reflow events (24). The reason why overexpansion worsens vessel condition and prognosis in STEMI patients may be attributed to the higher prevalence of unstable plaques, lipid pools, necrotic cores, and thrombi, which are easier to break and extrude through the deployed stent struts (25) during stent implantation or excessive postdilation; then, microemboli may be formed, leading to distal microvascular embolism and elevated obstructive resistance. The risk of distal embolism of high-pressure dilation in STEMI patients even prompts the intervention of distal protection devices, which has been shown to be effective in reducing the incidence of no-reflow events (26). Meanwhile, coronary dissection and coronary rupture are more easily caused by higher dilatation. Avoiding overexpansion makes more sense as complete restoration of myocardial perfusion is the major objective in the treatment of STEMI. However, the conclusion that an appropriate underexpansion between 70 and 80% does not affect prognosis may be potential of great significance, which can guide PCI strategy in the future.

The relationship between postdilation or stent expansion and hemodynamics is extremely controversial. A previous study showed that plenary postdilation and optimal stent expansion did not significantly increase the MR overall, and the index of microcirculatory resistance (IMR) increased in half of the patients but decreased in the other half at an individual level (17). Another study showed that MR remained elevated in over 30% patients postdilation; only patients with pain to wire time >6 h had a partial reduction in MR, while thrombotic burden and stent expansion were related to the increase in MR (2). The reason why MR or IMR is elevated postdilation may be attributed to microvascular injury and dysfunction led by inflation, NC balloon manipulation, or fragmentation and distal embolization of atherothrombotic material. However, another study in STEMI patients where IMR decreased postdilation may be attributed to the increase in coronary flow after the obstruction has been revascularized, which led to increased perfusion pressure and subsequent downstream relaxation of microcirculation (2). These results indicate that the response of microcirculation to stent expansion and postdilation was variable. We found that, overall, stent expansion did not linearly affect QFR or MR; this is consistent with previous studies that showed that QFR and MR did not change significantly after PCI, and that patients with lower baseline MR had a significant increase in MR, which may be caused by coronary manipulation (balloon predilation or thrombus aspiration) (14). However, we observed a nonsignificant trend of QFR increase along with stent expansion, which is also surprising. According to the model of FFR, it is expected that stent dilatation affects the vessel volume, blood flow velocity, and other parameters which affect the blood vessel function (6, 9). In addition, previous studies have confirmed that the QFR was positively correlated with stent expansion in patients undergoing nonemergency PCI (8). Focusing on the ambiguous relationship between QFR and stent expansion in STEMI patients, we found that no-reflow was prevalent and it is usually caused by distal embolism, which comes from unstable plaque and high thrombotic burden. Meanwhile, some studies have shown that the higher the stent pressure, the higher the chance of a non-reflow event (24). So we hypothesize that the relationship between QFR or vascular physiology benefit and the expansion of vessels with or without stents in STEMI patients may not be linear but a u-shaped curve. When underexpansion occurs, luminal dimension losses in the traditional ways result in insufficient distal blood flow and impaired blood flow reserve. A recent study showed stenosis and plaque > 70% predicts QFR </= 80% (27). However, when the vessels or stents undergo overexpansion, emboli, such as lipid pool and necrotic material, are squeezed out and may embolize the distal vessels, resulting in increased microcirculation resistance and a slow blood velocity. Moreover, due to the Bernoulli effect, the blood flow velocity flow through the enlarged lumen is further slowed down. The calculation formula shows that QFR positively correlates with blood flow velocity (6), so this is the reason for us to assume that QFR decreases when the expansion is too large. However, when the expansion degree is in an appropriate range, the distal embolization is not serious and the stenosis in the lesion stage is relatively ideal. This ideal state takes into account the thrombotic burden, the plaque burden, vascular physiology, and the traditional concept of coronary intervention. We will further expand the cases in the future and try to verify our hypothesis through modeling. Interestingly, subgroup analysis focusing on MR change showed that the MR deterioration group had a higher QCA stenosis rate, lower reference lumen CSA, higher stent expansion, higher MR before stenting, higher flow speed before stenting, and lower flow speed after stenting, which indicates that blood flows through this kind of vessels at a high speed before stenting; MR deteriorated after stent implantation and postdilation as STEMI lesions usually possess vulnerable plaques with lipid pool, high level of microinflammatory state, and high thrombus burden, which are susceptible to intracoronary manipulation. Therefore, the phenomenon that stent expansion can reduce flow speed is logical and consistent with the conclusion that overexpansion increases the incidence of no-reflow events. To explain the mechanism of no-reflow and flow speed, previous studies hypothesized that fragmentation of atherosclerosis material and distal embolization led by postdilatation caused further microvascular injury in the cases with increased MR (2, 28). We also measured the volume and ratio of plaque and found that necrotic plague in the over-expansion group is higher and the volume and ratio of calcified plague in the underexpansion group is higher. This founding further indicates the factors affecting stent expansion and consists with the consensus that calcification may lead to underexpansion, and a higher ratio of unstable (necrotic and lipidic) plague may lead to more risk of cardiovascular events. What's more, a substantial number of patients had slow or no-reflow before stenting in this study. In our follow-up study (29), we found that the no-/slow-reflow event guided by thrombolysis in myocardial infarction (TIMI) flow grade occurred in 38 (15.5%) of the 245 STEMI patients. It was quite a high rate, so the influence of slow reflow cannot be ignored. Studies have confirmed that slow or no-reflow phenomenon may influence QFR/MR (30) as the flow speed is included in the process of QFR/MR calculation. So we checked our data and performed a subgroup analysis between patients with and without a no-reflow event (Supplementary Table 1), and interestingly we found that QFR before stent (0.59 without no reflow vs. 0.77 with no-reflow, *p* = 0.04) and MRangio before stent (202 without no-reflow vs. 157 with no-reflow, *p* = 0.03) differ between the two groups. However, these results indicate that the original condition of vessels with no-reflow is better than that of vessels with reflow, which is contradictory to the expectation that slow flow or no-reflow may lead to an impaired QFR/MRangio. Focusing on this question, we further checked other parameters and found that patients with no-reflow events had a longer and larger size of stent, more stent expansion, larger reference CSA, and a similar plaque burden but a higher ratio of necrotic plaque (Supplementary Table 1). Thus, plaque and operation may play key roles, and we assume that QFR/MRangio and no-reflow may be the results of the interaction between the plaque and the operation. The relationship between no-reflow and QFR or MR is worthy to be discovered. However, QFR after stenting did not differ among the groups, which is consistent with a recent study that found that FFR values were not significantly different between optimal expansion and underexpansion (*p* = 0.23) in selected PCI (31). This may further correspond to the phenomenon that the appropriate underexpansion does not affect prognosis, although MR increases after PCI in STEMI patients, which consequently leads to a concept of tolerable underexpansion. Guided by this concept, the strategy of PCI in STEMI may evolve into a more precise pattern in the future. Although the finding that QFR/MR seemed not be linked to the extent of stent expansion is surprising, yet we actually observed a trend that QFR increases along with stent expansion, and an expanded sample size may help us understand the relationship between QFR and stent expansion in STEMI patients. Furthermore, due to the uniqueness and complexity of patients with high thrombus burden, it will be worthy to include patients undergoing nonemergency PCI or without high thrombus burden as a control group in the future. The strategy of pPCI taking into account vessel function and stent expansion will be more clear.

The current study had several limitations that may have influenced the observed results. Firstly, this study reflected the experience with a limited number of patients. Since IVUS is not routinely used in pPCI of STEMI in clinical work, the sample sizes of previous studies that focused on IVUS, vascular physiology, and STEMI are generally small (*n* = 117, Geng; *n* = 51, Luo, D) (27, 29). To increase the sample size, minimize the bias, and improve reliability, this study was designed as a multicenter clinical trial. We also selected a long inclusion time of 1 year. Secondly, vessel function was not assessed by the golden standard (FFR); rather, it was assessed by noninvasive QFR. Nevertheless, our results showed that the incidence of no-reflow and MACE in STEMI was associated with stent overexpansion, which highlights the necessity of avoiding this condition. Moreover, appropriate underexpansion could be tolerable in consideration of its noninferiority compared with optimal expansion in the short-term prognosis.

## Conclusions

In the present retrospective analysis based on the assessment of stent expansion using commercially available IVUS and QFR software, stent expansion appears to provide additional prognostic information. Appropriate stent underexpansion of 70 and 80% may be tolerable as it does not affect vessel function and short-term prognosis. Overexpansion and underexpansion of <70% should be avoided due to the higher risk for MACE and noreflow events. However, the small number of events reported does not allow us to draw definite conclusions. Therefore, further confirmatory research is needed on a larger number of patients to assess the predictive accuracy of stent expansion combined with QFR and IVUS for the precise improvement of PCI.

## Data Availability Statement

The original contributions presented in the study are included in the article/Supplementary Material, further inquiries can be directed to the corresponding authors.

## Ethics Statement

The studies involving human participants were reviewed and approved by Research Ethics Committee Guangdong General Hospital, Guangdong Academy of Medical Sciences. The patients/participants provided their written informed consent to participate in this study.

## Author Contributions

HD and ST conceived the study. XL, XY, XG, and JY collected the data. DL, SS, and BS re-examined the data. XG, YZho, and HD analyzed the data. XL wrote the manuscript. HD, ST, and JL reviewed and revised the manuscript. All authors contributed to the article and approved the submitted version.

## Funding

This study was funded by the National Key Research and Development Program of China, Grant (2016YFC1301202) and by Shanghai Jiao Tong University, Med-X Foundation Project, Grant (YG2021ZD04).

## Conflict of Interest

The authors declare that the research was conducted in the absence of any commercial or financial relationships that could be construed as a potential conflict of interest.

## Publisher's Note

All claims expressed in this article are solely those of the authors and do not necessarily represent those of their affiliated organizations, or those of the publisher, the editors and the reviewers. Any product that may be evaluated in this article, or claim that may be made by its manufacturer, is not guaranteed or endorsed by the publisher.
