# Supplementary material for: Microvascular and Prognostic Effect in Lesions With Different Stent Expansion During Primary PCI for STEMI: Insights From Coronary Physiology and Intravascular Ultrasound
Source: Front Cardiovasc Med. 2022 Mar 9;9:816387. doi: 10.3389/fcvm.2022.816387 (PMC8959302; doi:10.3389/fcvm.2022.816387)
Supplement: Supplementary file 1 [file Table_1.docx]

Supplement Table 1 Data on patients with and without no-reflow events

|  | Studied patients (n=87) | No-reflow (n=7) | Reflow (n=80) | p |
| --- | --- | --- | --- | --- |
|  |  |  |  |  |
| Reference Lumen CSA (mm2) | 9.9±4.0 | 9.38±4.3 | 9.95±4 | 0.02 |
| Minimum lumen CSA (mm2) | 3.5±2.6 | 4.3±2.7 | 3.4±2.6 | 0.11 |
| Plaque burden (%) | 79±5 | 78±7 | 80±5 | 0.18 |
| Plaque ratio (%) |  |  |  |  |
| Fibrotic | 65% | 64.2% | 65.1% | 0.72 |
| Necrotic | 24% | 25.8% | 23.2% | 0.02 |
| Lipidic | 9.6% | 9% | 9.9% | 0.31 |
| Calcified | 1.4% | 1% | 1.8% | 0.25 |
| Plaque volume (mm3) |  |  |  |  |
| Fibrotic | 6.66±2.2 | 6.57±2.1 | 6.67±2.8 | 0.3 |
| Necrotic | 2.5±1.1 | 2.68±0.9 | 2.41±1.1 | 0.03 |
| Lipidic | 0.99±0.02 | 0.92±0.04 | 1.02±0.07 | 0.07 |
| Calcified | 0.15±0.016 | 0.11±0.01 | 0.19±0.012 | 0.02 |
| Stent segment |  |  |  |  |
| Stent diameter(mm) | 3.2±0.7 | 3.5±0.5 | 3.1±0.8 | 0.04 |
| Stent length(mm) | 25±7 | 26±7 | 24±8 | 0.02 |
| Minimum stent CSA (mm2) | 6.9±2.4 | 6.7±1.7 | 7.6±5.5 | 0.68 |
| Stent expansion (%) | 82±24 | 86±34 | 81±23 | 0.01 |
| cQFR before stenting | 0.64 | 0.77±0.13 | 0.59±0.17 | 0.04 |
| MR before stenting (mm Hg*s/m) | 203.9 | 157±91 | 204.3±183 | 0.03 |
| Flow speed before stenting(cm/s) | 15.6 | 12.7±4.2 | 15.9±7.8 | 0.59 |
| cQFR after stenting | 0.93 | 0.98±0.01 | 0.93±0.08 | 0.12 |
| MR after stenting (mm Hg*s/m) | 268.5 | 348±43 | 246±69 | 0.01 |
| Flow speed after stenting(cm/s) | 17.4 | 8.9±2.6 | 18.4±6.3 | 0.01 |
