# Supplementary material for: Microvascular and Prognostic Effect in Lesions With Different Stent Expansion During Primary PCI for STEMI: Insights From Coronary Physiology and Intravascular Ultrasound
Source: Front Cardiovasc Med. 2022 Mar 9;9:816387. doi: 10.3389/fcvm.2022.816387 (PMC8959302; doi:10.3389/fcvm.2022.816387)
Supplement: Supplementary file 2 [file Table_2.docx]

| Supplement table 2 Multivariate analyses of stent expansion and the change of vessel functional diameters | | | | |
| --- | --- | --- | --- | --- |
| Multivariate analysis | HR | P | Confident interval | |
| **Stent expansion(per 1% increase)** |  |  |  | |
| △QFR | -0.003 | 0.19 | -0.008 – 0.002 | |
| △MR (mm Hg*s/m) | -3.69 | 0.57 | -3.3 – 10.2 | |
| △Flow speed (cm/s) | 0.24 | 0.41 | -0.27 – 0.50 | |
| **Optimal-expansion** |  |  |  | |
| △QFR | 0.14 | 0.31 | -0.09–0.26 | |
| △MR (mm Hg*s/m) | 22.4 | 0.35 | -33.6 – 35.9 | |
| △Flow speed (cm/s) | -0.7 | 0.19 | -2.1 – 1.9 | |
| **Over-expansion** |  |  |  | |
| △QFR | 0.19 | 0.22 | -0.10–0.55 | |
| △MR (mm Hg*s/m) | 26.6 | 0.86 | -96.8 –52.3 | |
| △Flow speed (cm/s) | 9.2 | 0.41 | -16.6 – 36.5 | |
| **Under-expansion** |  |  |  | |
| △QFR | -0.28 | 0.81 | -0.46 – 0.20 | |
| △MR (mm Hg*s/m) | 23.6 | 0.84 | | -35.1 – 36.2 |
| △Flow speed (cm/s) | 5.4 | 0.24 | | -8.1 – 19.1 |
